# Synthesis and properties of fluorescent 4′-azulenyl-functionalized 2,2′:6′,2″-terpyridines

**DOI:** 10.3762/bjoc.12.171

**Published:** 2016-08-11

**Authors:** Adrian E Ion, Liliana Cristian, Mariana Voicescu, Masroor Bangesh, Augustin M Madalan, Daniela Bala, Constantin Mihailciuc, Simona Nica

**Affiliations:** 1 “C. D. Nenitzescu” Institute of Organic Chemistry of the Romanian Academy, 202 B Splaiul Independentei, 060023, Bucharest, Romania; 2 Inorganic Chemistry Laboratory, Faculty of Chemistry, University of Bucharest, Str. Dumbrava Rosie, 020464, Bucharest, Romania; 3 Department of Organic Chemistry, Biochemistry and Catalysis, Faculty of Chemistry, University of Bucharest, Bd. Regina Elisabeta 4-12, Bucharest 030016, Romania; 4 “Ilie Murgulescu” Institute of Physical Chemistry of the Romanian Academy, Splaiul Independentei 202, 060021, Bucharest, Romania; 5 Department of Chemistry, Hazara University, Mansehra 21120, Pakistan; 6 Physical Chemistry Department, Faculty of Chemistry, University of Bucharest, Regina Elisabeta, no. 4-12, 030018, Bucharest, Romania

**Keywords:** azulene, fluorescence, metal binding, synthesis, terpyridine

## Abstract

4′-Azulenyl-substituted terpyridines were efficiently synthesized following the Kröhnke methodology via azulenylchalcone intermediates. These azulenyl-containing terpyridines showed fluorescent emission with a fluorescence quantum yield varying from 0.14, in the case of parent terpyridine, to 0.64 when methyl groups are grafted on the azulenyl seven-membered ring. According to the crystal structures and TDDFT calculations, different twisting of the aromatic constituents is responsible for the observed fluorescent behavior. The electrochemical profile contains one-electron oxidation/reduction steps, which can only be explained on the basis of the redox behavior of the azulene unit. The ability of the 4′-azulenyl 2,2′:6′,2″-terpyridine to bind poisoning metal cations was studied by UV–vis titrations using aqueous solutions of Hg(II) and Cd(II) chlorides as illustrative examples.

## Introduction

2,2′:6′,2″-Terpyridine derivatives are extensively used organic ligands in the field of supramolecular chemistry and materials science [1–4]. Besides the interesting supramolecular architectures, metal–terpyridine complexes have been investigated for their potential applications in catalysis, photovoltaic cells and biomedical science [5]. Special attention has been paid to fluorescence responses of metal–terpyridine compounds permitting access to new sensors for bioassays and in vivo imaging purposes [6]. The characteristics of the metal-containing assemblies depend on the electronic influence of the substituents attached to the terpyridine unit as well as to the metal ion [7]. While the number of publications concerning applications or investigations of inorganic–organic hybrid structures has increased enormously, comparably few fluorescent 4′-functionalized 2,2′:6′,2″-terpyridine derivatives have been reported [8]. It has been established that 2,2′:6′,2″-terpyridine has low quantum yield fluorescence [9] and significant emission can be achieved after specific modifications of the terpyridine core motif, especially by introducing conjugated moieties at the 4′-position [10–12]. In this context, the synthesis of tailored terpyridine derivatives with appropriate electron-donor/acceptor moieties may allow for a further improvement of their spectroscopic and electrochemical properties. Owing to its unique fluorescent and remarkable optical and redox properties, azulene proved to be an excellent building block for developing a large variety of materials ranging from NLO chromophores [13] to molecular switches [14–15] and liquid crystals [16] or high-conductance materials [17]. In contrast to most aromatic compounds which exhibit S_1_→S_0_ fluorescence under low excitation intensity, azulene shows fluorescence predominantly from the S_2_ excited state and only very weakly from S_1_ [18]. The control of the optical and electronic properties of the azulene-based materials can be finely tuned by careful selection of the substitution patterns. Moreover, the azulene moiety can easily be modified by the introduction of various functional groups owing to its ability to react with both nucleophilic and electrophilic reagents [19–21].

We have recently shown that 4′-azulenyl-substituted terpyridines can be successfully involved in the development of an efficient ruthenium catalyst for selective oxidation of both aliphatic and aromatic amines to nitriles [22]. The catalytic effectiveness of this ruthenium terpyridine complex was ascribed to the polarization effect of the azulene moiety attached at the terpyridine unit and it was sustained by comparison with analogues ruthenium complexes with unsubstituted or 4′-phenyl-substituted terpyridine.

In this contribution we report on the green synthesis and physicochemical investigations of the 4′-azulenyl-substituted terpyridines with particular interest on the fluorescence properties. The origin of the fluorescence emission will be also described by time-dependent density functional theory (TDDFT) calculations. The ability of the terpyridine compounds to bind poisoning metal cations was investigated by spectrophotometric titrations of methanolic 4′-azulenyl-substituted terpyridine solution with aqueous Hg^2+^ and Cd^2+^ solutions. Most importantly, these new terpyridine compounds not only show interesting properties, but also offer a great variety of potential synthetic modifications.

## Results and Discussion

### Synthesis and characterization

**Scheme 1:**
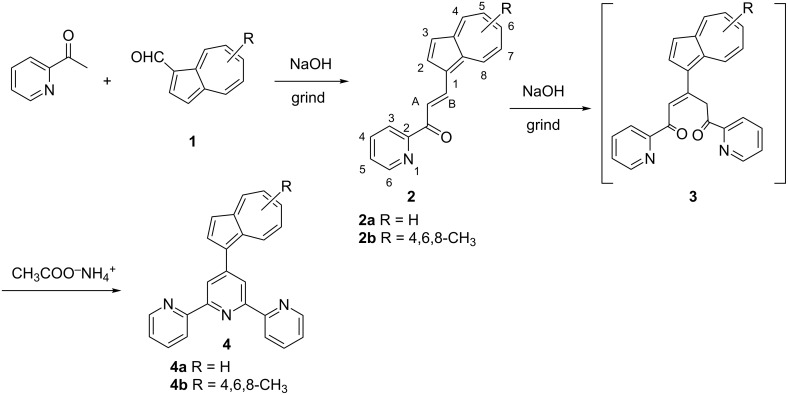
Synthesis of 4′-azulenyl substituted terpyridines.

The target compounds have been prepared following the Kröhnke-type synthetic methodology [23–24] starting from azulene carbaldehydes (**1**) [25] and 2-acetylpyridine (Scheme 1). In the first step, the 1-azulenyl-2′-azachalcone precursor **2** is formed via a Claisen–Schmidt aldol condensation. This reaction was performed in an environmentally friendly chemical manner, by grinding neat starting materials without the use of classical organic solvents [26]. The α,β-unsaturated ketone was formed in good yields, 72% for **2a** and 70% for **2b**, respectively using NaOH as base. The azulenyl-substituted chalcones are stable at room temperature for months, whereas at high temperature and pressure, they decompose very rapidly. Alternatively, the chalcones **2** can be isolated following the conventional synthetic route, namely the reaction of equimolar amounts of the azulene-carbaldehyde with 2-acetylpyridine in ethanol, at room temperature or, by microwave irradiation at 110 °C for 10 min in aqueous medium. In both cases, the desired α,β-unsaturated ketone **2** were isolated in similar yields (see Table 1).

**Table 1:** Synthesis of the 1-azulenyl-2’-azachalcone **2a**.

Entry	Conditions	Yield %

1	grind/NaOH/rt	72
2	EtOH/stirring/NaOH/rt	70
3	MW/H_2_O/NaOH/110 °C	67^a^
4	grind KOH/rt	75

^a^The microwave power was around 30 W.

It is worth mentioning here that the microwave-assisted reaction was performed in aqueous medium, an environmentally benign solvent. The other advantage of this last synthetic procedure is given also by the easy separation of the desired compounds, namely simple filtration and washing of the formed solid with alcohol. An improvement of the reaction yield could be achieved by replacing NaOH with KOH (Table 1, entry 4).

The formation of these intermediates is confirmed by ^1^H NMR spectroscopy which reveals two doublets at 8.6 and 8.3 ppm assigned to COC*H_A_*=C*H_B_* with large coupling constants (*J*_A,B_ = 15.2–15.6 Hz) indicative of the *trans*-double bond. In the case of compound **2b**, the ^1^H NMR spectrum showed downfield shifts for the β-olefin proton resonance (9.0 ppm) and upfield shifts for the signals assigned to the azulene moiety. This upfield shielding of the azulenyl protons is a consequence of the inductive effect of the electron-donating methyl groups present in **2b**. Further evidence for the structural assignment of **2a** is given by the presence of characteristic stretching vibrations at 1690, and 1654 cm^−1^ associated with the CO–C=C bond in the IR spectrum.

The subsequent grinding reaction of the azulene-functionalized azachalcone **2** with an equimolar amount of an inorganic base and 2-acetylpyridine affords brownish-red solid compounds. Any attempt to isolate this intermediate failed. The compound is very unstable and it decomposes very rapidly, both in solid and in solution. In accordance with previous reports [23,27] and with ESIMS analysis [28], we hypothesized that this corresponds to a diketone structure. This raw, freshly prepared compound is reacted with excess ammonium acetate in acetic acid under microwave irradiation at 160 °C for 5 minutes. The target terpyridines are isolated in satisfactory yields varying from 42% in the case of compound **4a** to 35% for **4b**, respectively. If the reaction is performed under refluxing conditions for 4–6 hours, the desired 4′-azulenyl substituted terpyridines are obtained as trace compounds, the yields of the reaction do not exceed 10%. Alternatively, the desired 4′-azulenylterpyridines can be isolated using a step-by-step reaction protocol, without the isolation and purification of the chalcone precursors when the overall yield of the reaction is less than 5% lower.

The ^1^H and ^13^C NMR spectroscopic data for both terpyridine compounds **4** are consistent with the proposed chemical structures. Comparison with the ^1^H NMR spectrum of the 4′-phenyl-2,2′:6′,2″-terpyridine analogue [27,29–30] evidences that the pyridine proton resonances are not significantly affected by the azulene-magnetic field. The 6,6″-pyr*H*, and 3,3″-pyr*H* (pyr = pyridine) resonances for both compounds, **4a** and **4b**, appear as multiplets at 8.70–8.67 ppm, similar to phenyl-substituted terpyridine compounds (Table 2, entries 1, 2 and 3).

**Table 2:** Reaction conditions for the synthesis of 1-azulenyl-2′-azachalcone **2a**.

Compound	3/5′-pyrH^a^	3,3″ 6,6″-pyrH^a^	2-azH^b^	3-azH^b^	5-azH^b^	7-azH^b^

2,2′:6′,2″-terpyridine^c^	7.93	8.62/8.69	–	–	–	–
4′-phenyl-2,2′:6′,2″-terpyridine^d^	8.69	8.81/8.66	–	–	–	–
**4a**	8.74	8.71–8.67	8.26	7.45	7.26	7.20
**4b**	8.52	8.70–8.67	7.73	7.37	7.10	7.04

^a^pyr: pyridine; ^b^az: azulene; ^c^Ref. [8]; ^d^Ref. [30].

Instead, the 3′,5′-pyrH resonance shifts upon azulene substitution, especially in the case of compound **4b**, when the upfield shielding is around 0.2 ppm. The observed shielding effect is caused by the electron-donating methyl groups attached to on the seven-membered azulenyl ring in terpyridine **4b**. These substituents are also responsible for the upfield shift of the azulene proton resonances, the most affected being the azulene protons at the 2- and 3-positions (Table 2).

**Figure 1:**
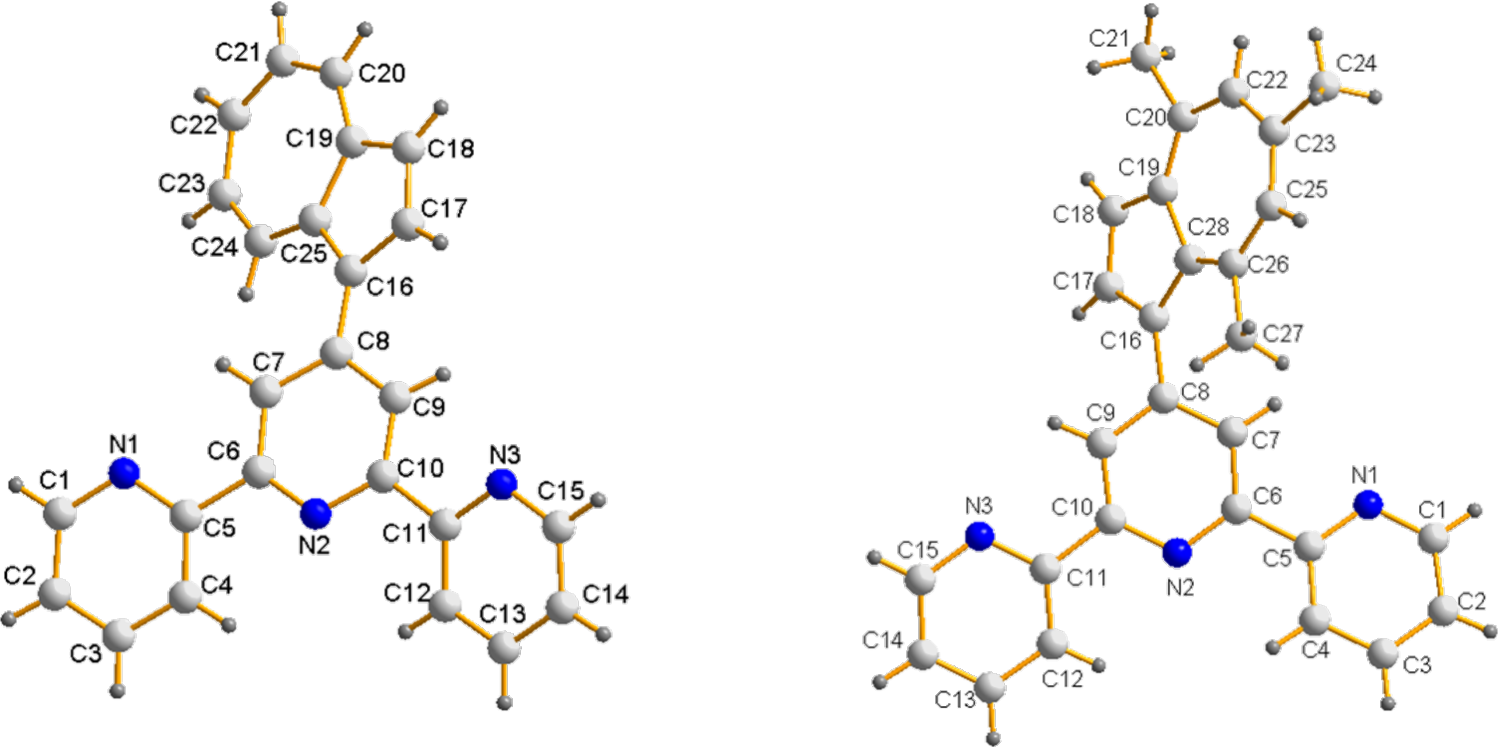
Molecular structure and numbering scheme of 4′-(1-azulenyl)-2,2′:6′,2″-terpyridine (**4a**, left) and 4′-(1-(4,6,8-trimethyl-azulenyl)-2,2′:6′,2″-terpyridine (**4b**, right).

Crystals suitable for X-ray diffraction could be isolated for both compounds by crystallization from methanol/dichloromethane/acetonitrile solutions upon slow evaporation of the solvents at room temperature. The crystal structures confirm the solution assignment for both compounds. The 4′-azulenyl-substituted terpyridine **4a** crystallized in the orthorhombic *P*2_1_*cn* space group, whereas **4b** crystallized in the monoclinic *C*2/*n* space group. The molecular structures are depicted in Figure 1. More crystallographic data for compounds **4a** and **4b** are contained in the cif files (Supporting Information File 1 and Supporting Information File 2) and the CCDC files [31].

The three pyridine rings are in *transoid* arrangement about the interannular C–C bonds as previously described for similar terpyridine compounds [8,27,29]. The interannular C–C bonds are 1.484(5) and 1.487(5) Å in **4a**, respectively and 1.490(5) and 1.491(5) Å in **4b**. The pyridine rings are not coplanar, the torsion angles between the two terminal pyridines and the central pyridine ring are different within the herein described compounds. The twisting of the pyridine rings is more evident in **4a** where the torsion angles between the mean planes of the terminal pyridine rings and the central one are of 20.2 and 13.6°, respectively. This twisting is higher than that of 4’-phenylterpyridine or its substituted derivatives [27]. Furthermore, the azulene connected to the terpyridine fragment is also twisted about the interannular bond such that its mean plane makes an angle of 25.1° with the central pyridine ring, comparable to that of 4′-(p-aminophenyl)terpyridine (27.5°) [32] and 4′-(p-bromophenyl)terpyridine (22.83°) [33]. Instead, in the case of **4b**, the twisting of the pyridine rings of the terpyridine fragment is smaller; the terminal pyridine rings are distorted about the interannular C–C bond by torsion angles of 3.5 and 11.3°, similar with the corresponding distortion observed for 4′-phenyl and 4′-anilino-substituted terpyridines [27,32]. The trimethyl-substituted azulenyl fragment is not coplanar with the terpyridine unit, being twisted about the interannular C–C bond by a torsion angle of 51.3°, much larger than in the case of **4a**.

**Figure 2:**
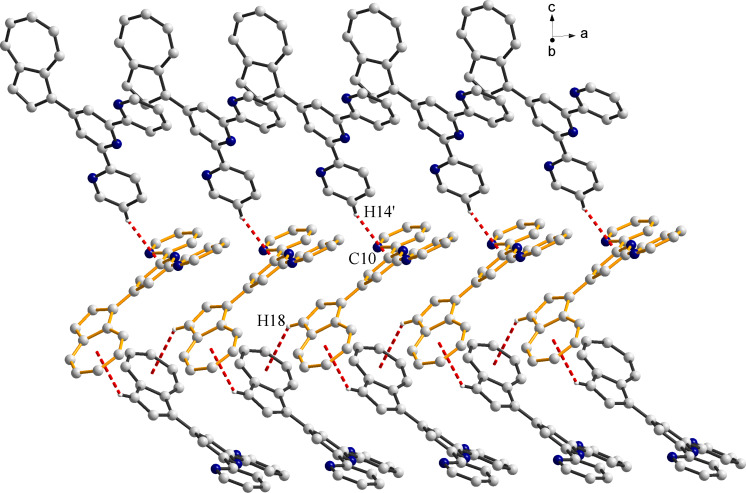
Packing diagram for **4a** showing the π–π stacking and CH–π interactions between the pyridine rings and the azulenyl moieties. Only the hydrogen atoms involved in hydrogen bonding interactions are shown.

**Figure 3:**
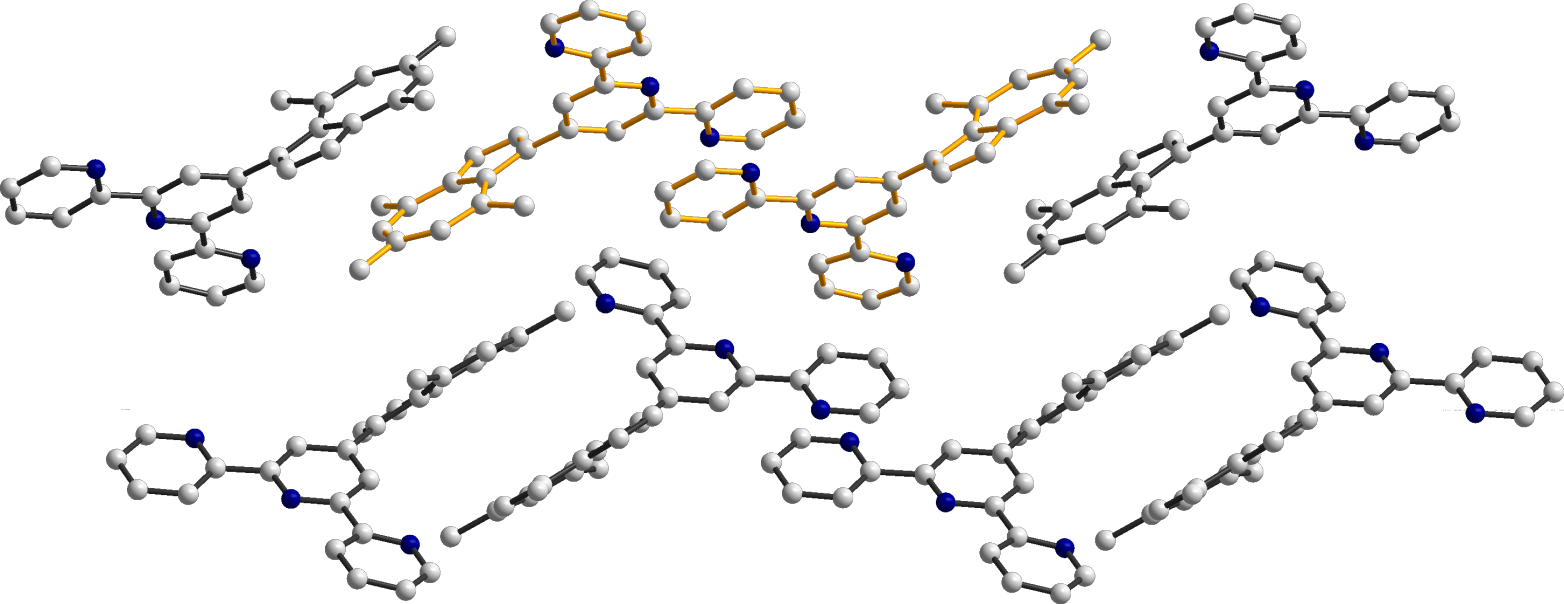
Packing diagram for **4b** showing the π–π stacking between the pyridine rings. Hydrogen atoms are omitted for clarity.

In both cases, interesting supramolecular interactions are observed in the crystal packing, via the azulene moieties and the terpyridine groups. In the crystal of **4a,** the molecules are organized in columns running along the crystallographic *a* axis by π–π stacking interactions (3.39–3.64 Å). In the neighboring columns the molecular units exhibit a herringbone arrangement (Figure 2). This organization is sustained by CH–π interactions (H18∙∙∙centroid and H14′∙∙∙C10 contacts are 3.00 and 2.89 Å, respectively; symmetry code: ’ = −0.5 + x, −0.5 + y, 0.5 − z). Instead, the analysis of the packing diagrams in **4b** (Figure 3) shows that only the terpyridine fragments are involved in π–π interactions (3.56–3.66 Å). The separation between the neighboring azulenyl moieties is higher than 3.85 Å.

### Photophysical properties

**Figure 4:**
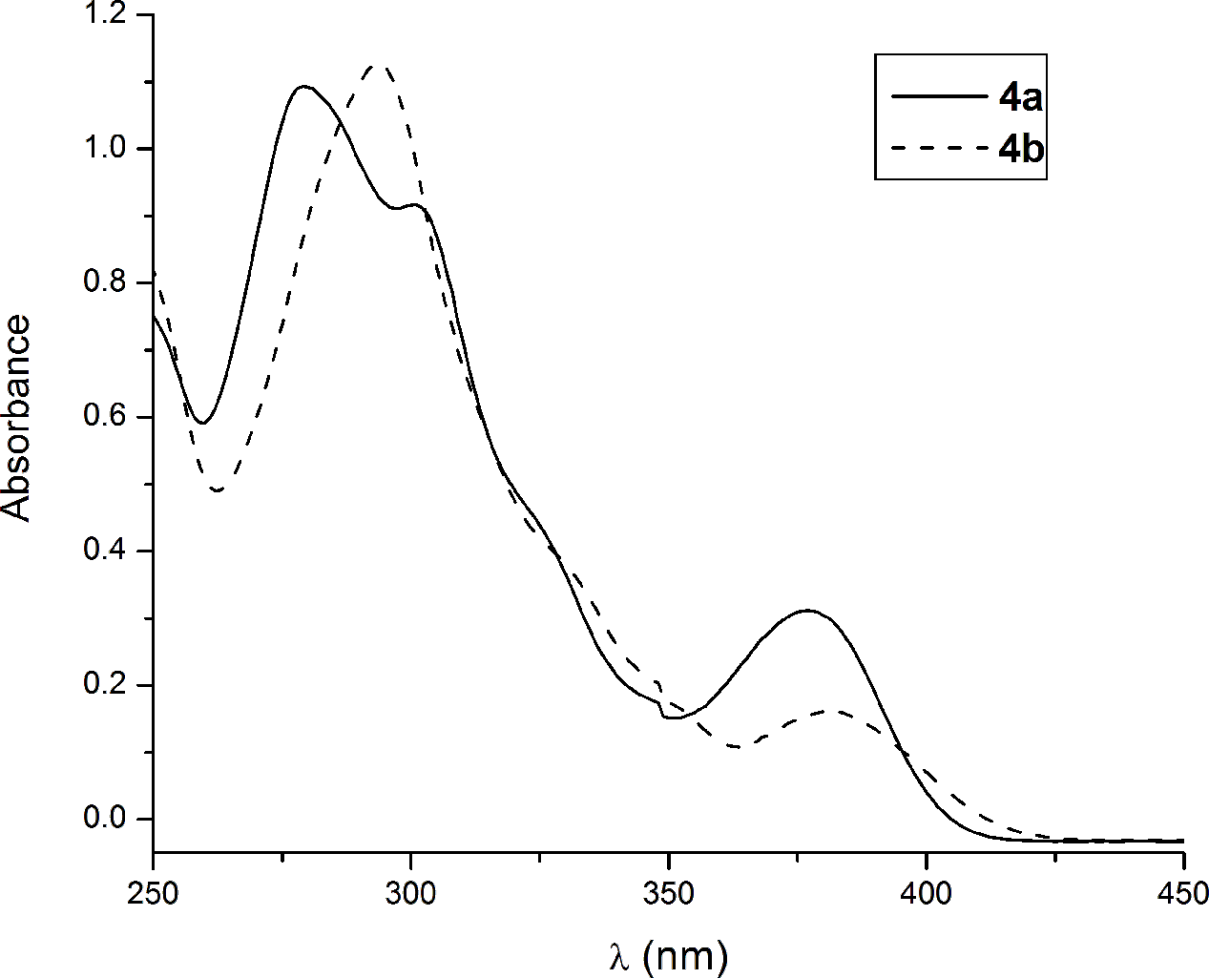
Absorption spectra of the azulene-containing terpyridine, **4a** and **4b** in CH_2_Cl_2_ solution at room temperature.

The photophysical properties of the azulene-containing terpyridines, **4a** and **4b** have been investigated by absorption and emission spectroscopy in dichloromethane solution. The 2,2′:6′,2″-terpyridine moiety is an excellent chromophore with an absorption maximum at 279 in dichloromethane solution [8]. Upon 4′-substitution with a phenyl group the longest-wavelength absorption is not affected, only the fluorescence emission is shifted bathochromically by only 3 nm. The UV–vis spectrum of the 4′-azulenyl-substituted terpyridine **4a** resembles the absorption maximum of the terpyridine core found below 300 nm, but the longest-wavelength absorption maximum is shifted to 377 nm. The absorption bands of the azulene-containing terpyridines are influenced by the azulenyl substituents, especially the π–π* transition band. Compared to **4a**, the presence of the electron donating groups in **4b** caused a pronounced bathochromic shift of the π–π* transition band, while the longest-wavelength absorption maximum is less affected (Table 3, Figure 4).

**Table 3:** Absorption and fluorescence maxima of 2,2′:6′,2″-terpyridine (tpy) derivatives in dichloromethane at room temperature.

Entry	λ_Abs_/nm (log ε)	λ_fls_/nm (Φ)

2,2′:6′,2″-tpy^a^	279.5 (4.30)	337 (0.02)
4′-phenyl-tpy^a^	278 (4.52)	340 (0.33)
**4a**	279 (4.58), 300 (sh), 377 (4.02)	435 (0.14)/530 (sh)
**4b**	294 (4.61), 381 (2.73)	427 (0.64)/522 (sh)

^a^Taken from [8].

The results of TDDFT calculations showed that the longest wavelength absorption maximum originates from a π–π transition centered on the azulene moiety but having some azulene→central pyridine charge transfer as electrons from HOMO and LUMO+1 orbitals (Figure 6) are involved. The HOMO (orbitals 94 and 107 for **4a** and **4b** respectively in Figure 6) is localized only over azulene moiety, while the LUMO+1 (orbitals 96, 108 for **4a** and **4b**, respectively) is an orbital delocalized over the azulene moiety and the central pyridine ring. On the other hand both DFT calculated (vide infra) and X-ray structures show that the azulene moiety is twisted from the central pyridine ring to a greater extent in **4b**. It becomes clear that the substitution on the azulene moiety tends to break the azulene/central pyridine planarity and possibly reduce the azulene→central pyridine charge transfer effect, hence affecting the absorption maximum.

**Figure 5:**
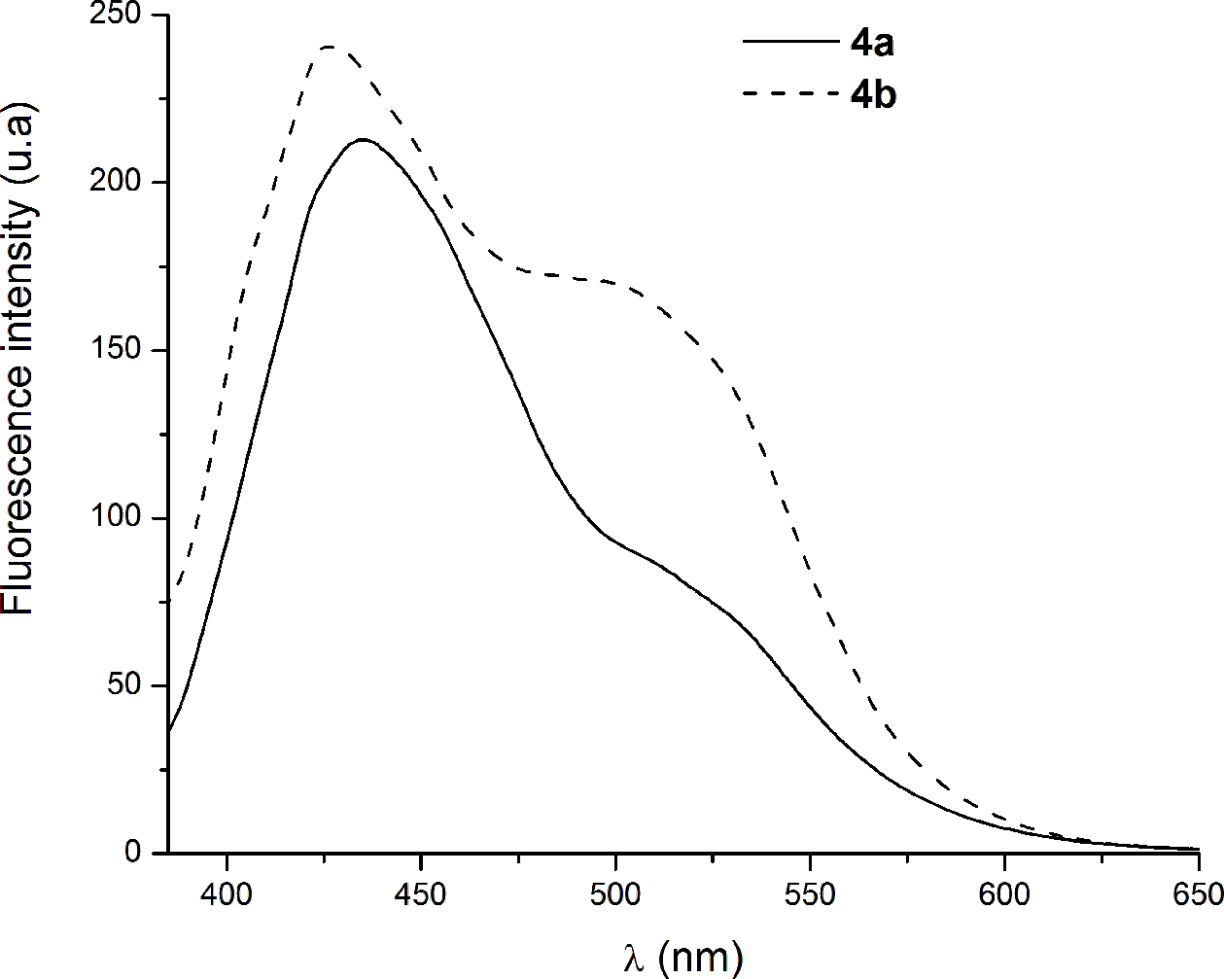
Emission spectra of the azulene-containing terpyridine, **4a** and **4b** in CH_2_Cl_2_ solution (2.59 × 10^−5^ M) at room temperature.

Both compounds showed fluorescent emission upon excitation at wavelengths corresponding to their absorption maximum. The fluorescence spectra are shown in Figure 5. In the case of **4a**, by excitation with 375 nm, a dual fluorescence emission is observed at 435 nm and 530 nm, respectively (Table 3). According to literature reports on azulene fluorescent behavior, the first emission wavelength is attributed to the S_2_→S_0_ transition, whereas the second emission can be a consequence of the S_1_→S_0_ transition [18]. By comparison the fluorescence intensity of **4b**, not only increases but it also shows a strong hypsochromic shift for both transitions with the emission wavelengths at 427 nm and 522 nm, respectively (Figure 5, Table 3). Moreover, the fluorescence quantum yields of these compounds were greatly affected by the azulenyl substitution. While, 4′-azulenylterpyridine showed a medium fluorescence emission (0.14), the methyl substitution markedly improved the quantum yield to 0.64 (Table 3). Because the recording of the fluorescence spectra was performed under the same conditions for both compounds, the changes in the emission efficiency can be explained by the presence of a different conjugation path. According to the crystal structure, the torsion angle between the azulene plane and the central pyridine ring of the terpyridine fragment is larger than the corresponding one observed in compound **4a**. Therefore, in these lines can be observed that proper substitution of the azulenyl moiety can influence the emission behavior and it may be changed from dominant S_2_→S_0_ fluorescence to either dual fluorescence or to dominant S_1_→S_0_ fluorescence.

### Theoretical calculations

#### Methods

The geometries of **4a** and **4b** with initial atomic coordinates taken from X-ray crystal structure were optimized with B3LYP functional and 6-31G(d) basis. The resulting molecular structures are shown in Figure S1 (Supporting Information File 3) and the corresponding internal coordinates in Table S1 (Supporting Information File 3). In general, the calculated bond lengths, bond angles, and orientation of the atoms relative to each other as reflected by dihedral angles agree well with the crystal structure. The orientation of the azulene moiety with respect to the pyridine rings is in agreement with the crystallographic data as shown by the torsion angles of carbon atoms number 20 and 23 relative to the plane of the central pyridine ring. The three pyridine rings are calculated to have higher coplanarity in comparison to the crystal structure, showing the easy rotation of pyridine rings about the interanullar axis in gas phase, in contrast to constraints imposed by the solid crystal. The electronic excitations and oscillator strengths for the herein described compounds **4a** and **4b** were calculated with time-dependent density functional theory (TDDFT) utilizing the following functionals: B3LYP [34–37], Coulomb-attenuated B3LYP (CAMB3LYP) [38], hybrid TPSS (TPSSH) [39], and M06 with double Hartree–Fock exchange (M062X) [40]. Of these four functionals, B3LYP and CAMB3LYP are hybrid functionals with 20% Hartree–Fock exchange content, the later one incorporated with enhanced Hartree–Fock (HF) exchange in the long-distance ranges in order to correctly describe the charge transfer (CT) states. TPSSH is a hybrid version of the metaGGA TPSS functional containing 10% HF exchange. M062X is a metaGGA functional with “double” HF exchange content (54%) meant to produce correct long-range behavior. A number of Pople type gaussian basis functions were used to see the effects of basis size, and of polarization and diffuse functions. Presently, the results with the largest basis used (6-311+G(d,p)) are presented in Table 4. All calculations were performed with GAMESS(US) [41] and ORCA [42] suites of quantum chemical codes.

**Table 4:** 6-311+G(d,p) vertical excitations in eV (oscillator strength) for **4a** and **4b** in increasing energy order.

Transition	B3LYP		CAMB3LYP	
	**4a**	**4b**	**4a**	**4b**

I	2.270 (0.006)	2.488 (0.008)	2.320 (0.008)	2.570 (0.010)
II	3.251 (0.194)	3.146 (0.075)	3.534 (0.171)	3.539 (0.137)
III	3.262 (0.047)	3.163 (0.103)	4.126 (0.025)	4.133 (0.010)
IV	3.947 (0.141)	3.953 (0.168)	4.269 (0.602)	4.213 (0.646)
V	4.209 (0.201)	4.234 (0.097)	4.541 (0.273)	4.393 (0.081)
VI	4.234 (0.273)	4.238 (0.281)	4.548 (0.279)	4.511 (0.304)
VII	4.594 (0.414)	4.463 (0.362)		

	TPSSH		M062X	
	**4a**	**4b**	**4a**	**4b**

I	2.284 (0.005)	2.475 (0.007)	2.436 (0.013)	2.659 (0.014)
II	3.049 (0.002)	2.922 (0.001)	3.709 (0.234)	3.695 (0.186)
III	3.113 (0.216)	2.988 (0.140)	4.105 (0.007)	4.087 (0.003)
IV	3.783 (0.094)	3.880 (0.103)	4.355 (0.469)	4.288 (0.410)
V	4.110 (0.382)	4.122 (0.391)	4.633 (0.185)	4.568 (0.246)
VI	4.146 (0.044)	4.300 (0.133)		
VII	4.470 (0.376)	4.414 (0.333)		

#### Absorption spectra

The lower energy absorption band for **4a** centered at 377 nm (3.29 eV) and that for **4b** at 381 nm (3.25 eV) is due to excitation to close lying second and/or third excited electronic states as shown by oscillator strengths listed for transitions II and III in Table 4. The experimental excitation energy values are closely reproduced by functionals B3LYP and TPSSH, and overestimated by CAMB3LYP and M062X. The absorption shoulder located at 300 nm (4.13 eV) in **4a** is reproduced in the matching energy range by B3LYP (transitions IV, V, VI), CAMB3LYP (transition IV), and TPSSH (transition V). M062X does not show any matching absorption with significant oscillator strength to be assigned to the experimental absorption shoulder observed for **4a**. The higher energy absorption band centered at 279 nm (4.44 eV) for **4a** is assigned to the following transitions calculated with different functionals: B3LYP (VII at 4.59 eV), CAMB3LYP (closely lying V, VI at 4.541 and 4.548 eV respectively), TPSSH (VII at 4.470 eV), and M062X (IV at 4.355 eV). Clearly TPSSH gives the closest value to the experimentally recorded one. Regarding the observed higher energy absorption band of **4b** centered at 294 nm (4.22 eV), the visual comparison of the experimental absorption spectra of **4a** and **4b** (Figure 4) shows that the 300 nm (4.13 eV) shoulder of **4a** morphs into the 294 nm (4.22 eV) peak of **4b**, while 279 nm (4.44 eV) peak of **4a** completely disappears in **4b**. The B3LYP, CAMB3LYP and TPSSH functionals show an enhanced oscillator strength for the transitions matching to the 300 nm (4.13 eV) experimental band of **4b** (IV for B3lYP, CAMB3YLP; V for TPSSH), while all three functionals fail to diminish for **4b** the oscillator strengths of transitions corresponding to the 279 nm (4.44 eV) peak of **4a**. At this point it can be concluded that the longer wavelength absorption is attributed to electronic transitions to the second excited state localized in the azulene-fused ring. The shorter wavelength absorption is assigned to the transition to the fourth excited state comprised of excitation occurring on the azulene ring mixed with some azulene→terpy charge transfer.

#### Emission spectra

The observed S_2_→S_0_ emission for **4a** at 435 nm (2.85 eV) closely matches with energy gaps of the two close lying electronic transitions II and III obtained with the B3LYP and TPSSH functionals. In the case of **4a**, the calculated energies corresponding to this gap are 3.251 eV (transition II), and 3.262 eV (transition III) with B3LYP functional, and 3.049 eV (transition II) and 3.133 eV (transition III) with TPSSH. Concerning **4b**, the same gaps are 3.146 eV (II/B3LYP), 3.163 eV (III/B3LYP), 2.922 eV (II/TPSSH), and 2.988 eV (III/TPSSH), comparable to the experimental observed 2.90 eV (427 nm) emission. Thus, the B3LYP and TPSSH values compare well with observed emissions of both **4a** and **4b** in contrast with the much higher values obtained with CAMB3LYP and M062X functionals for transition II in both **4a** and **4b**. The observed longer wavelength S_1_→S_0_ emission at 530 nm (2.34 eV) for compound **4a** closely matches with the energy gap corresponding to transition I calculated to be 2.270 (B3LYP), 2.284 (TPSSH), 2.320 (CAMB3LYP) and 2.436 (TPSSH) eV. The same good correlation between experimental 2.38 eV (522 nm) and calculated values (2.488 (B3LYP), 2.475 (TPSSH), 2.570 (CAMB3LYP) and 2.659 (MO62X)) were obtained for the S_1_→S_0_ emission of **4b**.

#### Orbital origin of spectra

**Figure 6:**
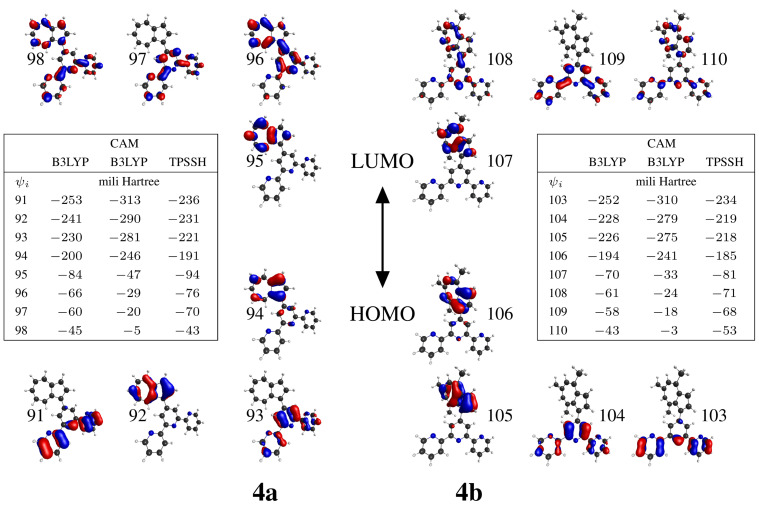
Selected Kohn–Sham orbitals and orbital energies for **4a** and **4b**, obtained with three different functionals and 6-311+G(d,p) basis.

The most important Kohn–Sham orbitals (ψ_i_) playing an active role in the calculated electronic transitions are shown in Figure 6 along with their energies.

The nature of the excited state(s) accessed through electronic transitions are listed in Table 4 is a contribution of various occupied→virtual excitations, the most dominant of which in terms of highest A^2^ values (square of calculated excitation amplitude) are given in Table S2 (Supporting Information File 3). The first excited electronic state arises predominantly from excitation of an electron in the highest occupied molecular orbital (HOMO) to the lowest unoccupied orbital (LUMO), the transition labeled as I in Table 4 and Table S2 (Supporting Information File 3). As obvious from the orbital shapes shown in Figure 6, it is azulene-centered valence transition (VT) involving π-electrons of this moiety. The oscillator strength of I is too weak to play a role in absorption, however, it is responsible for the S_1_→S_0_ emission. Close lying electronic excited states resulting from transitions II and III are dominated by HOMO→LUMO+1 excitation (azulene-centered valence transition, accompanied by some charge transfer (CT) from the azulene to the central pyridine ring), with relatively minor contribution from HOMO→LUMO+2 excitation (CT from the azulene to the terpyridine moiety). The transitions II and III are responsible for the lower energy absorption around 377 nm (**4a**) and 381 nm (**4b**) and for S_2_→S_0_ emission in the experimental spectra. Regarding the absorption shoulder around 300 nm observed in the experimental spectrum of **4a**, it was shown above that matching calculated transitions are IV, V, VI (B3LYP), IV (CAMB3LYP), and V (TPSSH). The orbital origin of these transitions is not consistent for these functionals. According to B3LYP these correspond to terpyridine→azulene CT, and terpyridine→terpyridine VT. TPSSH shows it to be terpyridine→terpyridine VT/azulene→azulene VT and also some azulene→central pyridine CT character. The high-energy absorption maximum involves transitions like terpy→terpy, terpy→azulene, azulene→terpy, though different functionals are not consistent in depicting the contributing ratios of these various components of electronic transitions.

#### Electrochemical properties

**Figure 7:**
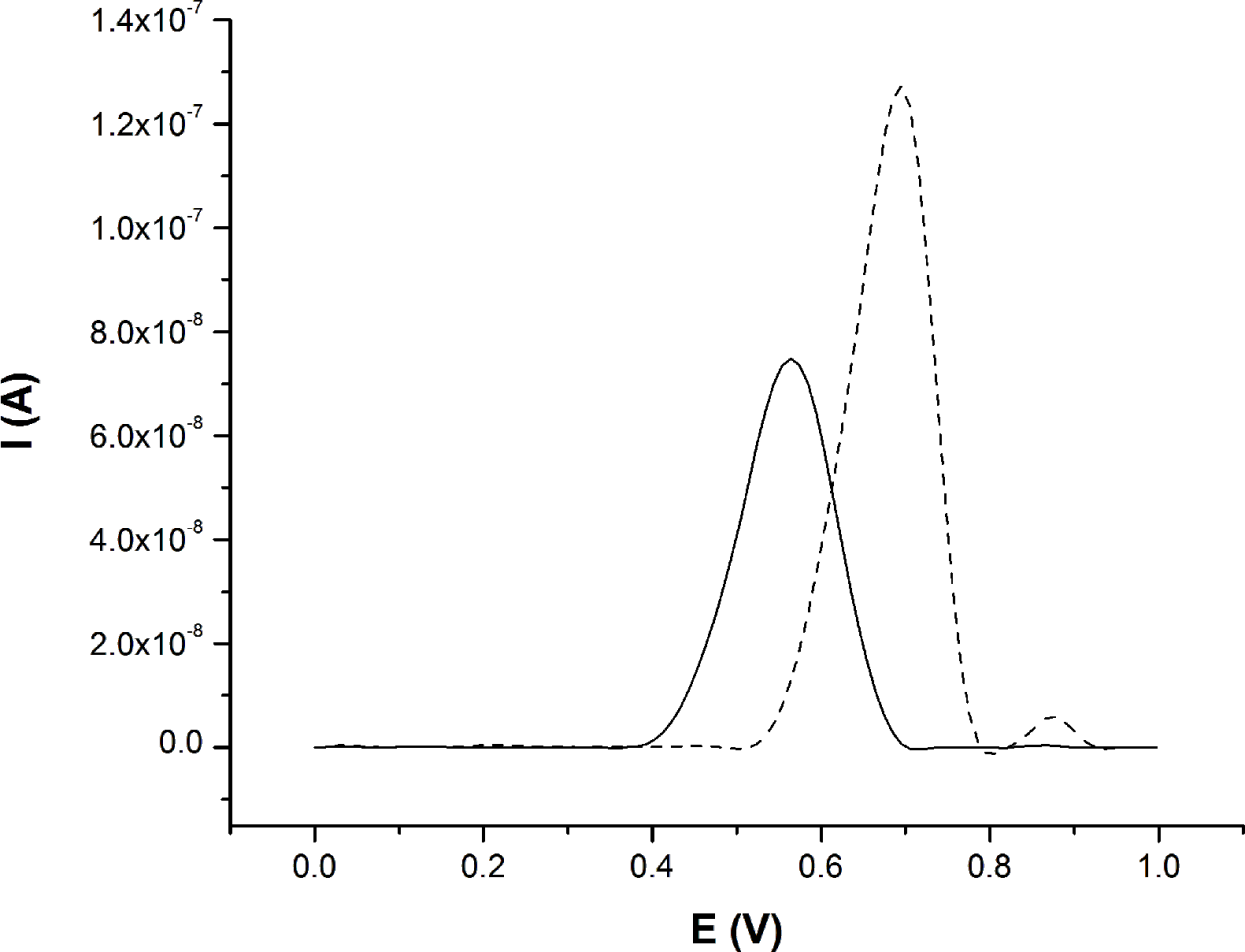
DPV-traces (with baseline correction) of 0.5 mM solutions of **4a** (solid) and **4b** (dash) in DMF, with SP = 10 mV and MA = 50 mV in the 0.0 V to 1.0 V potential range.

The redox properties of **4a** and **4b** were investigated using cyclic voltammetry (CV) and differential pulse voltammetry (DPV) in dimethylformamide, in the 0.0–1.0 V potential range. Both compounds exhibited similar CV profiles with two oxidation peaks at 0.1 V/s scan rate. The azulenyl-substituted terpyridine **4a** exhibited one quasi-reversible oxidation wave at around 0.09 V and another shoulder-like at 0.69 V. When methyl groups are introduced in the azulene unit (**4b**) both anodic peaks shift to positive potential values, at 0.14 V and 0.74 V, respectively. Thus, the oxidation of **4b** occurs harder owing to the stabilized 6π-electron tropylium cation due to the electron-donating effect of the methyl groups. In both cases, the more anodic peak was tentatively assigned to the formation of the cation radical of the azulene moiety. The DPV experiments confirmed that stabilization of the tropylium cation in the case of **4b**, the oxidation potential being shifted anodically at 0.70 V as compared to 0.56 V for **4a** (Figure 7).

For each compound the CV experiments were performed for two cycles. On the first positive ongoing scan a unique anodic peak appeared at around 0.66 V for **4a** and 0.72 V for **4b.** On the second positive ongoing scan a new anodic peak at ca. 0.09 V for **4a** and 0.14 V for **4b** was observed. This explains why in DPV experiment only the higher value of the anodic peak was observed. For the negative region, there is no counter peak in the 1.0 V to 0.0 V potential ranges.

#### Reactivity towards Hg^2+^ and Cd^2+^ complex formation

**Figure 8:**
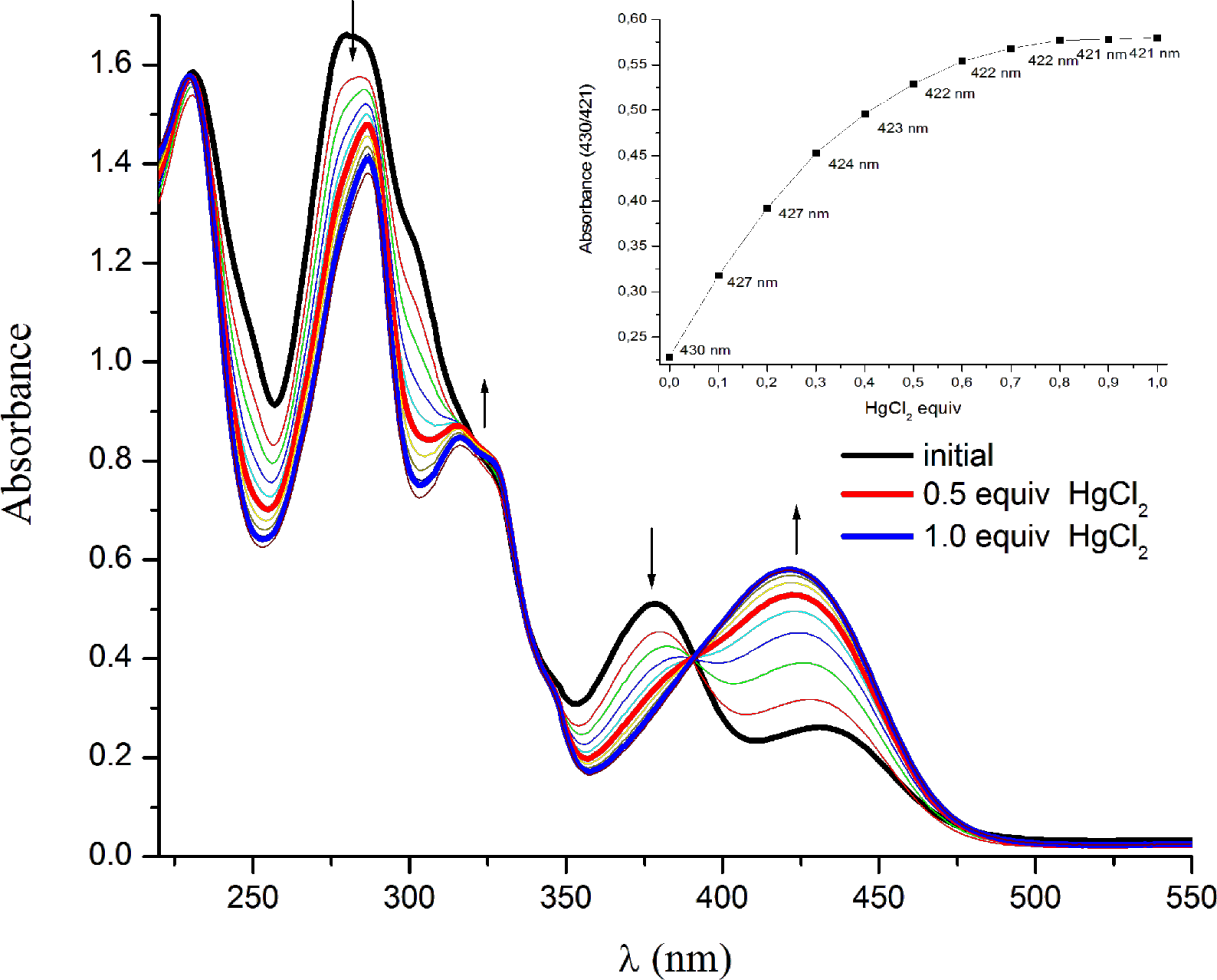
Absorption spectra of a 4.26 mM solution of **4a** in methanol upon titration with an aqueous HgCl_2_ solution (0–1.0 equivalents). Inset shows the visible absorption changes upon mercury binding.

**Figure 9:**
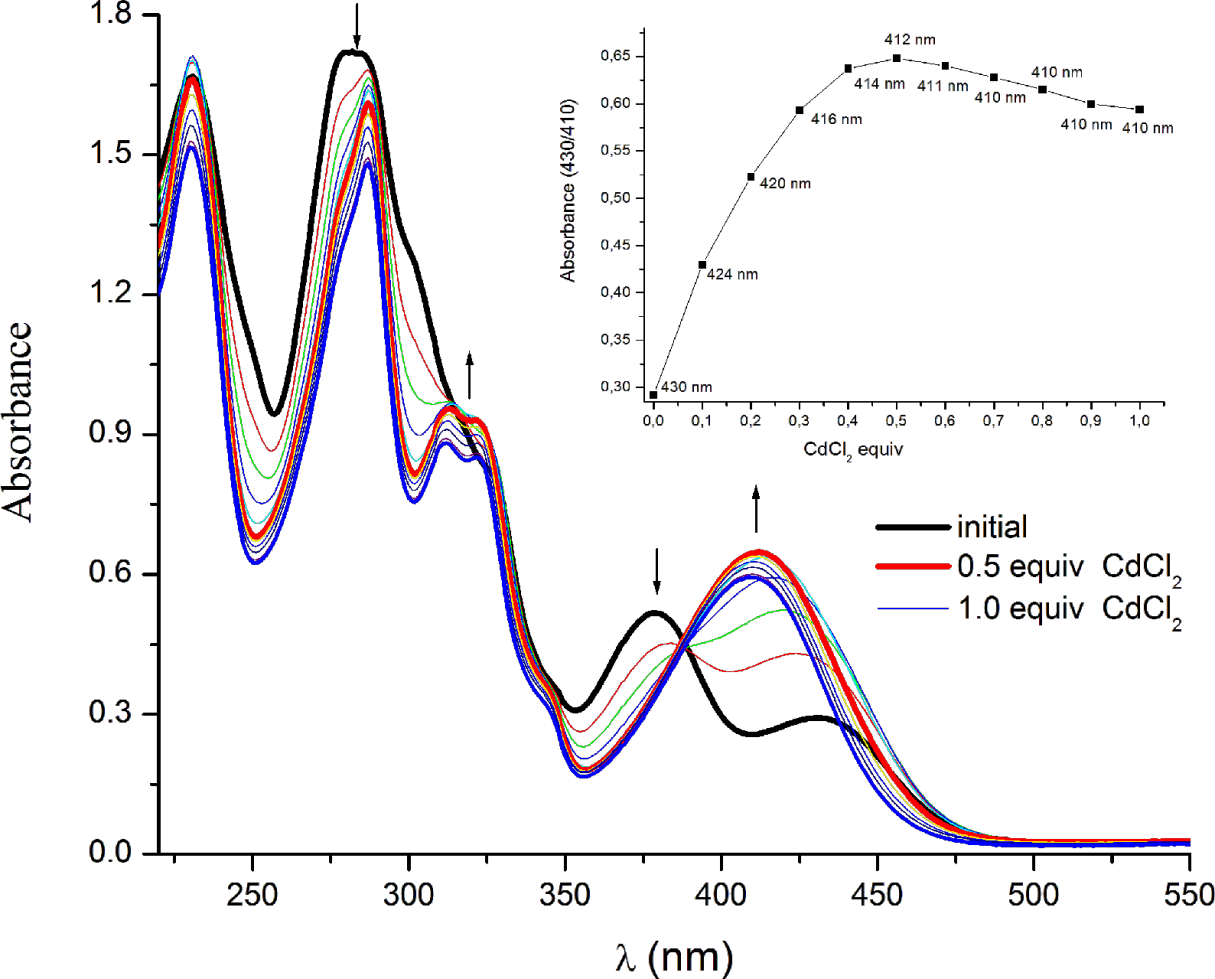
Absorption spectra of a 4.26 mM solution of **4a** in methanol upon titration with CdCl_2_ aqueous solution (0–1.0 equivalents). Inset shows the visible absorption changes upon cadmium binding.

The 2,2′:6′,2″-terpyridine derivatives are known to form stable complexes with transition metals by generating the corresponding [M(terpyridine)_2_]^2+^ or [M(terpyridine)]^2+^ complexes. This ability was exploited in the development of colorimetric “naked-eye” chemosensors for poisoning Hg(II) ions from drinking water in the presence of other metal pollutant competitors [43]. Therefore, based on the known colorful azulene-derivatives, we undertook preliminary experiments involving terpyridine **4a** for possible selective detection of poisoning metal ions. As representative examples, we have chosen Hg(II) and Cd(II) metal ions. UV–visible titration was performed by adding increasing amounts of a metal chloride aqueous solution into a methanol solution of ligand **4a** (4.26 mM, Figure 8 and Figure 9). The initial spectrum of **4a** in methanol reassembles the absorption bands discussed previously in dichloromethane, but shows also an additional absorption band at 431 nm (lg ε = 3.73) due to the possible formation of weak hydrogen bonds in this solvent [44]. For both metal ions similar absorption features were observed. Upon addition of HgCl_2_ solution, the absorption maxima located at 378 nm and 431 nm are merging into a new band at 421 nm upon mercury complex formation. A progressive decreasing of the band located at 283 nm and the concomitant appearance of a new band around 315 nm were observed (Figure 8). It appears clearly from the titration, that no additional absorption change is observed upon introduction of more than 0.5 equiv of HgCl_2_ (inset graph in Figure 8) and it can be said that a 2:1 complex is formed. The straight slope and its saturation at a ratio of 0.5 indicate a high binding constant which cannot be determined from this set of data.

To assess the terpyridine-metal complex formation, ^1^H NMR titration of **4a** with HgCl_2_ has been performed. The addition of 0.25 equivalents of the mercury salt to a solution of **4a** in CD_3_OD (38 mM) caused the formation of a precipitate that it is insoluble in the deuterated methanol. Further addition of 0.25 equivalents of HgCl_2_ afforded a pale yellow solution with a brownish precipitate on the bottom of the NMR tube. After evaporation of this solvent, the solid was re-dissolved in DMSO-*d*_6_. The comparison with the ^1^H NMR spectrum of free **4a** in the same deuterated solvent shows that the chemical shifts of all terpyridine and azulene protons are downfield shifted upon mercury complex formation (Figure S2, Supporting Information File 3). The most affected protons are H-6/6” and H-3’/5’, these being very sensitive to the coordination mode of the terpyridine ligand. The H-6/6” protons which are positioned next to the nitrogen atom of the lateral pyridine ring are downfield shifted upon mercury coordination by around 0.2 ppm. The most affected are the H-3’/5’ protons of the central pyridine unit that are deshielded by 0.3 ppm upon mercury complex formation. Significant downfield shifts were observed also for all the azulene protons. Overall, a symmetric structure can be assigned, corresponding to a [M(terpyridine)_2_]^2+^ structural motif.

A similar visible-change profile was observed in the case of CdCl_2_ titration, but here the analysis of the titration curve showed a different profile (Figure 9). First, a closer look at the resulting UV–vis spectrum after cadmium(II) complex formation shows that the absorption maximum is more hypsochromically shifted (412 nm) as compared to the previous case. The analysis of the absorption intensity showed that more than 0.5 equivalents of the metal chloride cause a decreasing of the absorption band reaching a constant value close to one equivalent with a 1:1 complex formation.

Both titrations show an isosbestic point (390 nm), suggesting that only two species (the uncomplexed tpy and the metal-complexed tpy) are present during the titration process. According to the observed saturation, it can be concluded that both M(terpyridine)^2+^ and M(terpyridine)_2_^2+^ are formed based on the metal ion.

## Conclusion

Novel 4′-azulenyl-substituted terpyridines were efficiently synthesized following the Kröhnke methodology. The azulenylchalcone intermediates were conveniently synthesized using grind or microwave-assisted aldol condensation procedures. The target terpyridine compounds exhibit fluorescence emission upon excitation at the corresponding absorption maximum. The fluorescence quantum yield is influenced by the azulenyl substitution from 0.14 in the case of the parent 4′-azulenyl-2,2′:6′,2″-terpyridine to 0.64 for the trimethyl substituted azulenylterpyridine. According to the crystal structures, different twisting was observed that might be responsible for the observed fluorescent profile. The solid-state crystal packing showed π–π stacking interactions between the pyridine rings in face-to-face or T-shape orientation, completed by slipped-off π–π stacked azulenyl moieties according to their polarization vectors. TDDFT calculations showed that the fluorescence properties are determined by azulene-centered valence-state excitation and azulene→terpyridine charge-transfer transitions. Both compounds exhibit electrochemical behavior with one-electron oxidation/reduction steps, which can only be explained on the basis of the redox behavior of the azulene unit. The UV–vis titration studies proved that 4′-azulenyl-2,2′:6′,2″-terpyridine can bind poisoning Hg(II) and Cd(II) metal ions in aqueous environment, but with no clear color change as it has been expected.

## Experimental

### Materials and instruments

All reagents for performing the chemical reactions were used as received. CH_2_Cl_2_ for fluorescence measurements was purchased from Merck. Dimethylformamide for cyclic voltammetry measurements was purchased from Sigma-Aldrich and kept over molecular sieves. Column chromatography was carried out on 40–63 mesh silica gel and aluminum oxide. Analytical thin-layer chromatography was performed on Merck silica gel 60 F254 plates. Visualization was accomplished with UV light and preliminary fluorescence emission was observed with 365 nm light on TLC spots. Starting azulene-1-carboxaldehydes were obtained following Vilsmeier reaction [25]. Melting points were determined with a Koehler Automatic Melting Point Range apparatus (K90190). Elemental analyses were performed with Perkin Elmer CHN 240B analyzer. NMR spectra were recorded in deuteriochloroform (CDCl_3_) containing TMS as internal standard with a Bruker Avance DRX 400 (^1^H: 400 MHz, ^13^C: 100.62 MHz) instrument; chemical shifts (δ) are expressed in ppm, and *J* values are given in Hz; Az denotes azulene protons. The splitting patterns are indicated as s, singlet; d, doublet; t, triplet; m, multiplet; td, triplet of doublets. Mass spectrometry was performed using a Varian 1200L Triple Quadrupole LC/MS/MS spectrometer by direct injection in ESI mode. Titrations were performed as constant host titrations (4.26 mM in methanol) at room temperature by the addition of aliquots of the respective metal chloride stock solution in methanol (working solution obtained by dilution with water). UV–vis spectra were recorded after each addition on a Varian Cary 100 spectrophotometer using 1 cm quarts cells and methanol/water as solvent. The fluorescence emission and excitation spectra were recorded with a Jasco FP-6500 spectrofluorometer equipped with a 150 W Xenon lamp. The excitation wavelength was 375 nm for **4a** and 380 nm for **4b** for working concentrations of 2.59 × 10^−5^ M. The fluorescence quantum yield was determined by comparison of diluted quinine bisulfate solution in 0.1 N H_2_SO_4_ (0.55 absolute quantum yield) [45]. Electrochemical measurements were carried out on a potentiostat-galvanostat system AutoLabPGStat 12, controlled by GPES (general purpose electrochemical system) electrochemical interface for Windows (version 4.9.007). Three electrodes in an one-compartment cell (10 mL) were used in all experiments. A platinum disk electrode (Metrohm, 3 mm in diameter) served as working electrode. The counter electrode was a Pt wire of large area. All experimental potentials were referred to Ag wire (Metrohm), used as quasi-reference electrode. The electrochemical measurements were carried out in anhydrous dimethylformamide containing 0.1 M tetrabutylammonium perchlorate (TBAP) as supporting electrolyte. The solutions containing the electroactive species and the supporting electrolyte were purged with argon for 15 minutes in order to remove the oxygen, and low pressure inert gas atmosphere was maintained above the solution during the electrochemical experiments. Microwave-assisted reactions were performed with a BIOTAGE Initiator reactor.

X-ray diffraction measurements were performed on a STOE IPDS II diffractometer, operating with Mo Kα (λ = 0.71073 Å) X-ray tube with graphite monochromator. The structures were solved by direct methods and refined by full-matrix least squares techniques based on F^2^ [46]. The non-H atoms were refined with anisotropic displacement parameters. Atomic scattering factors were taken from the international tables for X-ray crystallography. Hydrogen atoms were included but not refined. Calculations were performed using SHELX-2014 crystallographic software package. Drawings of the molecules were performed with the program Diamond 3. A summary of the crystallographic data and the structure refinement are given below.

Crystal data for compound **4a**: C_25_H_17_N_3_, *M* = 359.41 g∙mol^−1^, orthorhombic, *P*2_1_*cn*, *T* = 200 K, *a* = 6.0110(4), *b* = 9.5419(6), *c* = 31.230(3) Å, α = β = γ = 90°, *V* = 1791.2(2) Å^3^, *Z* = 4, μ(Mo Kα) = 0.080 mm^−1^, F(000) = 752, R_1obs_ = 0.0441, wR_2obs_ = 0.1008, R_1all_ = 0.0859, wR_2all_ = 0.1398, GoF = 1.077, largest difference peak and hole: 0.218/−0.235 e∙A^−3^.

Crystal data for compound **4b**: C_28_H_23_N_3_, *M* = 401.49 g∙mol^−1^, monoclinic, *C*2/*n*, *T* = 293 K, *a* = 34.707(3), *b* = 8.7500(7), *c* = 14.8991(14) Å, α = γ = 90°, β = 105.906(8), *V* = 4351.4(7) Å^3^, *Z* = 8, μ(Mo Kα) = 0.073 mm^−1^, F(000) = 1696, R_1obs_ = 0.0577, wR_2obs_ = 0.0993, R_1all_ = 0.1881, wR_2all_ = 0.1438, GoF = 0.889, largest difference peak and hole: 0.138/−0.162 e∙A^−3^.

### Synthesis

**(*E*)-3-(Azulen-1-yl)-1-(pyridin-2-yl)prop-2-en-1-one (2a): Route A.** A neat mixture of 2-acetylpyridine (121.0 mg, 0.11 mL, 1.0 mmol), 1-azulencarboxaldehyde (156 mg, 1.0 mmol) and NaOH (40.0 mg, 1.0 mmol) were placed in a mortar and grinded for 10–15 minutes while a green-brownish solid is formed. The solid compound is washed with ether and the crude product was purified by column chromatography on silica gel (2% EtOH/CH_2_Cl_2_) to give compound **2a** (187 mg, 0.72 mmol, 72%) as a green-brownish solid; IR (ATR) ν_max_: 3404, 3050, 2978, 1690, 1654, 1561, 1494, 1393 cm^−1^; ^1^H NMR (CDCl_3_, 400 MHz, δ in ppm) 8.76 (d, *J* = 6.6 Hz, C*H*_Ph_-3, 1H), 8.74 (d, *J* = 10.0 Hz, 1H, C*H*_Az_-8), 8.66 (d, *J* = 15.2 Hz, 1H, C*H_A_*=), 8.46 (d, *J* = 4.4 Hz, 1H, C*H_A_*_z_-2), 8.33 (d, *J* = 15.6 Hz, 1H, C*H_B_*=), 8.32 (d, *J* = 9.2 Hz, 1H, C*H_Az_*-4), 8.23 (d, *J* = 8.0, 1H, C*H*_Ph_-6), 7.87 (td, *J* = 8.0, 2.0 Hz, 1H, C*H*_Ph_-4), 7.69 (t, *J* = 9.8 Hz, 1H, C*H_Az_*-6), 7.49–7.46 (m, 1H, C*H*_Ph_-5), 7.45 (d, *J* = 4.4 Hz, 1H, C*H_Az_*-3), 7.37 (t, *J* = 10.0 Hz, 1H, C*H_Az_*-7), 7.30 (t, *J* = 9.8 Hz, 1H, C*H_Az_*-5); ^13^C NMR (CDCl_3_, 100.6 MHz, δ in ppm) 189.3 (*C*O), 155.1 (Cq), 148.7 (*C*H_Ph_-3), 145.1 (*C*q), 140.1 (*C*q), 139.0 (*C*H_Az_-6), 137.5 (*C*H_Az_-4), 136.9 (*C*H_A_=), 136.3 (*C*H_Ph_-4), 135.6 (*C*H_Az_-2), 134.3 (*C*H_Az_-8), 126.4 (*C*H_Az_-7), 126.2 (*C*H_Az_-5), 125.8 (*C*q), 125.6 (*C*H_Az_-3), 122.8 (*C*H_B_=), 120.4 (*C*H_Ph_-5), 117.1 (*C*H_Ph_-6); Anal. calcd for C_18_H_13_NO: C, 83.37; H, 5.05; N, 5.29; found: C, 83.3; H, 4.8; N, 5.3; MS (ESI^+^, 0.1% NH_3_, *m*/*z*, %): 242 (10%), 260 (MH^+^, 100%), 439 (25%), 502 (10%).

**Route B.** To a stirred solution of azulen-1-carboxaldehyde (156.0 mg, 1.0 mmol) in ethanol (15 mL) was added 2-acetylpyridine (121.0 mg, 0.11 mL, 1.0 mmol) followed by the addition of NaOH (40.0 mg, 1.0 mmol). The reaction mixture was stirred at room temperature for 10–15 min. After that, diethyl ether (30 mL) was added and the formed precipitate filtered off. After column chromatography on silica gel as mentioned above, the desired chalcone was isolated (180 mg, 69.5%).

**Route C.** Azulen-1-carboxaldehyde (156.0 mg, 1.0 mmol), 2-acetylpyridine (121.0 mg, 0.11 mL, 1.0 mmol) and NaOH (40.0 mg, 1.0 mmol) were suspended in water (1.5 mL) in a microwave vial of 2 mL. The reaction mixture was introduced in a Biotage apparatus and irradiated for 15 min at 110 °C. After column chromatography on silica gel with 2% ethanol in dichloromethane chalcone **2a** was isolated (174 mg, 67%).

**(*E*)-1-(Pyridin-2-yl)-3-(4,6,8-trimethylazulen-1-yl)prop-2-en-1-one (2b):** This compound was prepared following Route A described above for chalcone **2a** by grinding neat 2-acetylpyridine (121 mg, 0.11 mL, 1.0 mmol) with 4,6,8-trimethylazulene-1-carbaldehyde (198.0 mg, 1.0 mmol) and NaOH (40 mg, 1.0 mmol). The crude product was purified by column chromatography (2% EtOH/CH_2_Cl_2_) to give compound **2b** (212.0 mg, 0.7 mmol, 70%) as a red-brownish solid; IR (ATR) ν_max_: 3420 (br), 3042, 2980, 2864, 1690, 1563, 1492, 1351 cm^−1^; ^1^H NMR (CDCl_3_, 400 MHz, δ in ppm) 9.00 (d, *J* = 15.6 Hz, 1H, C*H_A_*=), 8.74 (d, *J* = 8.4 Hz, 1H, C*H*_Ph_-3), 8.22 (d, *J* = 4.4 Hz, 1H, C*H*_Az_-2), 8.20 (d, *J* = 8.0, 1H, C*H*_Ph_-6), 8.13 (d, *J* = 15.2 Hz, 1H, C*H_B_*=), 7.85 (td, *J* = 7.6, 1.6 Hz, 1H, C*H*_Ph_-4), 7.46–7.43 (m, 1H, C*H*_Ph_-5), 7.40 (s, 2H, C*H*_Az_-5/7), 7.30 (d, *J* = 4.8 Hz, 1H, C*H*_Az_-3), 3.18 (s, 3H, C*H*_3_-8), 2.86 (s, 3H, C*H*_3_-6), 2.61 (s, 3H, C*H*_3_-4); Anal. calcd for C_21_H_19_NO: C, 83.69; H, 6.35; N, 4.65; found: C, 83.7; H, 6.5; N, 4.2.; MS (ESI^+^, *m*/*z*, %): 302 (MH^+^, 100), 367 (15), 423 (25%).

**4′-(1-Azulenyl)-2,2′:6′,2″-terpyridine (4a):** A neat mixture of chalcone **2a** (1.0 mmol), 2-acetylpyridine (2.0 mmol) and NaOH or KOH (2.0 mmol) are grinded in a mortar for around 10 min while a brick colored solid is formed. The resulting crude compound is introduced in a microwave vial together with ammonium acetate (1.6 g, 20.0 mmol) and acetic acid (2.0 mL) and irradiated for 30 min at 160 °C. The resulting mixture is diluted with dichloromethane and purified by alumina column chromatography (5% petroleum ether/CH_2_Cl_2_) to give the title compound **4a** (150.0 mg, 0.41, 42%) as blue-violet solid; mp 186–188 °C; λ_max_ (log ε, CH_2_Cl_2_): 279 (4.58), 301 (sh), 377 (4.02); ^1^H NMR (CDCl_3_, 400 MHz, δ in ppm) 8.78 (d, *J* = 9.6 Hz, 1H, C*H*_Az_-8), 8.74 (s, 2H, C*H_tpy_*-3’/5’), 8.71–8.67 (m, 4H, CH*_tpy_*-3/3”-6/6”), 8.36 (d, *J* = 9.6 Hz, 1H, C*H*_Az_-4), 8.26 (d, *J* = 4.0 Hz, 1H, C*H*_Az_-2), 7.84 (td, *J* = 7.6, 1.6 Hz, 2H, CH*_tpy_*-4/4”), 7.62 (t, *J* = 9.8 Hz, 1H, C*H*_Az_-6), 7.45 (d, *J* = 4.0 Hz, 1H, C*H*_Az_-3), 7.32–7.30 (m, 2H, CH*_tpy_*-5/5”), 7.26 (t, *J* = 9.6 Hz, 1H, C*H*_Az_-7), 7.20 (t, *J* = 9.6 Hz, 1H, C*H*_Az_-5); ^13^C NMR (CDCl_3_, 100.6 MHz, δ in ppm) 156.6 (*C*q), 155.6 (*C*q), 149.1 (*C*H*_tpy_*-3/3”), 147.0 (*C*q), 142.7 (*C*q), 138.4 (*C*H_Az_-6), 137.5 (*C*H_Az_-4/2), 136.7 (*C*H*_tpy_*-4/4”), 136.0 (*C*q), 135.4 (*C*H_Az_-8), 128.5 (*C*q), 124.5 (*C*H_Az_-7), 124.0 (*C*H_Az_-5), 123.6 (*C*H*_tpy_*-5/5”), 121.4 (*C*H*_tpy_*-6/6”), 121.3 (*C*H*_tpy_*-3’/5’), 118.0 (*C*H_Az_-3); Anal. calcd for C_25_H_17_N_3_: C, 83.54; H, 4.77; N, 11.69; found: C, 83.7; H, 4.5; N, 12.0.; MS (ESI^+^, *m*/*z*, %): 360 (MH^+^, 100), 361 (25%).

**4′-(4,6,8-Trimethylazulen-1-yl)-2,2′:6′,2″-terpyridine (4b):** The title compound **4b** was isolated following the above described procedure using 4,6,8-trimethylazulene-1-carbaldehyde (198.0 mg, 1.0 mmol). Compound **4b** (140 mg, 0.35 mmol, 35%) was isolated as violet-black solid. mp >300 °C; λ_max_ (log ε, CH_2_Cl_2_): 294 (4.61), 381 (2.73); ^1^H NMR, (CDCl_3_, 400 MHz, δ in ppm) 8.70–8.67 (m, 4H, C*H_tpy_*-3/3”-6/6”), 8.52 (s, 2H, C*H_tpy_*-3’/5’), 7.86 (td, *J* = 7.6, 1.6 Hz, 2H, C*H_tpy_*-4/4”), 7.73 (d, *J* = 4.0 Hz, 1H, C*H*_Az_-2), 7.37 (d, *J* = 4.0 Hz, 1H, C*H*_Az_-3), 7.33–7.30 (m, 2H, C*H_tpy_*-5/5”), 7.10 (s, 1H, C*H*_Az_-7), 7.04 (s, 1H, C*H*_Az_-5), 2.92 (s, 3H, C*H*_3_-8), 2.63 (s, 3H, C*H*_3_-6), 2.60 (s, 3H, C*H*_3_-4); ^13^C NMR (CDCl_3_, 100.6 MHz, δ in ppm) 156.7 (*C*q), 154.4 (*C*q), 151.9 (*C*q), 149.2 (*C*H*_tpy_*-3/3”), 147.3 (*C*q), 146.3 (*C*q), 145.9 (*C*q), 138.3 (*C*q), 136.7 (*C*H*_tpy_*-4/4”), 136.5 (*C*H_Az_-2), 132.0 (*C*q), 130.1 (*C*q), 129.5 (*C*H_Az_-7), 127.7 (*C*H_Az_-5), 123.6 (*C*H*_tpy_*-5/5”), 122.8 (*C*H*_tpy_*-3’/5’), 121.4 (*C*H*_tpy_*-6/6”), 115.4 (*C*H_Az_-3), 29.4 (*C*H_3_-6), 28.5 (*C*H_3_-4), 25.6 (*C*H_3_-8); Anal. calcd for C_28_H_23_N_3_: C, 83.76; H, 5.77; N, 10.47; found: C, 83.4; H, 5.2; N, 10.8; MS (ESI^+^, *m*/*z*): 402 (100, MH^+^), 403 (30%).

## Supporting Information

File 1 Computational details and the optimized geometries of the two 4′-azulenyl-2,2′:6′,2″-terpyridine compounds **4a** and **4b** and overlay of the ^1^H NMR spectra of free 4′-azulenyl-2,2′:6′,2″-terpyridine and the corresponding mercury(II) complex.

File 2 Crystallographic information file of compound **4a** (CCDC 1420841).

File 3 Crystallographic information file of compound **4b** (CCDC 1420842).
