# Supplementary material for: Synthesis and properties of fluorescent 4′-azulenyl-functionalized 2,2′:6′,2″-terpyridines
Source: Beilstein J Org Chem. 2016 Aug 11;12:1812–25. doi: 10.3762/bjoc.12.171 (PMC5082674; doi:10.3762/bjoc.12.171)
Supplement: File 1 — Computational details and the optimized geometries of the two 4′-azulenyl-2,2′:6′,2″-terpyridine compounds 4a and 4b and overlay of the 1H NMR spectra of free 4′-azulenyl-2,2′:6′,2″-terpyridine and the corresponding mercury(II) complex. [file Beilstein_J_Org_Chem-12-1812-s001.pdf]

Supporting Information

for

Synthesis and properties of fluorescent 4′-azulenyl-functionalized 2,2′:6′,2″-terpyridines

Adrian E. Ion[1,2], Liliana Cristian[1,3], Mariana Voicescu[4], Masroor Bangesh[5], Augustin M. Madalan[2], Daniela Bala[6], Constantin Mihailciuc[6] and Simona Nica[1]*

Address: [1]"C. D. Nenitzescu" Institute of Organic Chemistry of the Romanian Academy, 202 B Splaiul Independentei, 060023, Bucharest, Romania, [2]Inorganic Chemistry Laboratory, Faculty of Chemistry, University of Bucharest, Str. Dumbrava Rosie, 020464, Bucharest, Romania, [3]Department of Organic Chemistry, Biochemistry and Catalysis, Faculty of Chemistry, University of Bucharest, Bd. Regina Elisabeta 4-12, Bucharest 030016, Romania, [4]"Ilie Murgulescu" Institute of Physical Chemistry of the Romanian Academy, Splaiul Independentei 202, 060021, Bucharest, Romania, [5]Department of Chemistry, Hazara University, Mansehra 21120, Pakistan and [6]Physical Chemistry Department, Faculty of Chemistry, University of Bucharest, Regina Elisabeta, no. 4-12, 030018, Bucharest, Romania

Email: Simona Nica - simona.nica@ccocdn.ro

*Corresponding author

[§]Tel: + 4021 316 79 00; Fax: +4021 312 16 01; second email address: simonanica@yahoo.com

Computational details and the optimized geometries of the two 4′-azulenyl-2,2′:6′,2″-terpyridine compounds 4a and 4b and overlay of the ^{1}H NMR spectra of free 4′-azulenyl-2,2′:6′,2″-terpyridine and the corresponding mercury(II) complex

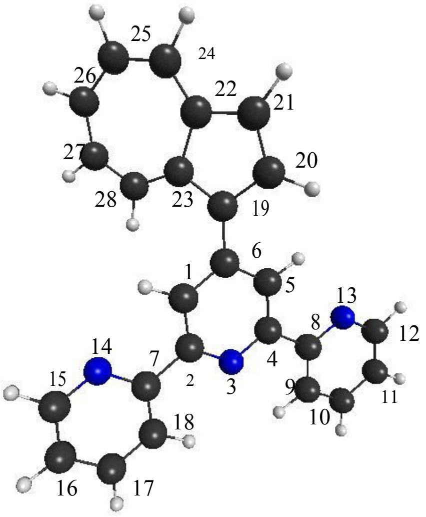

4a

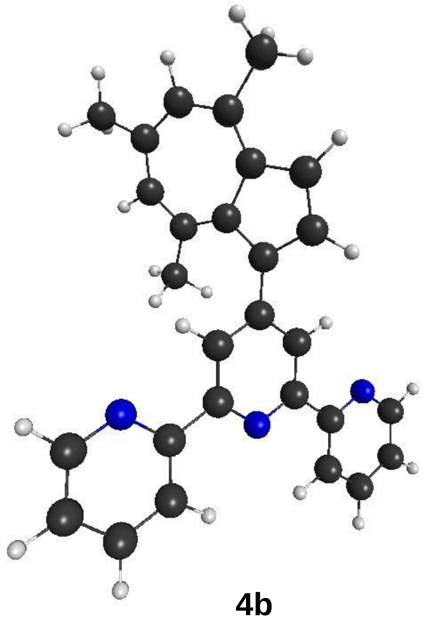

4b

Figure S1: Geometries of **4a** and **4b** optimised at B3LYP/6-31G(d) level.

Table S1: Bond lengths (pm), α, bond angles and τ, dihedrals (°). Atom numbering is according to atomic labels in Figure S1.

	**4a**	**4b**		**4a**	**4b**
$C_{1,2}$	140.0	139.9	$\alpha_{2,1,6}$	119.7	119.7
$C_{2,3}$	134.2	134.3	$\alpha_{7,2,3}$	117.0	117.1
$C_{3,4}$	134.4	134.3	$\alpha_{14,7,2}$	116.8	116.8
$C_{4,5}$	139.8	140.1	$\alpha_{15,14,7}$	118.2	118.2
$C_{5,6}$	140.5	140.2	$\alpha_{16,15,14}$	123:7	123.7
$C_{6,1}$	140.4	140.2	$\alpha_{17,16,15}$	117.9	117.9
$C_{4,8}$	149.2	149.2	$\alpha_{18,17,16}$	119.0	119.0
$C_{8,9}$	140.3	140.3	$\alpha_{7,18,17}$	118.9	118.9
$C_{9,10}$	139.2	139.2	$\alpha_{14,7,18}$	122.2	122.1
$C_{10,11}$	139.5	139.5	$\alpha_{8,4,3}$	116.9	117.0
$C_{11,12}$	139.6	139.6	$\alpha_{9,8,4}$	120.9	121.1
$C_{12,13}$	133.5	133.5	$\alpha_{10,9,8}$	118.9	118.9
$C_{13,8}$	134.6	134.6	$\alpha_{11,10,9}$	119.0	119:0
$C_{2,7}$	149.3	149.2	$\alpha_{12,11,10}$	117.9	117.9
$C_{7,14}$	134.6	134.6	$\alpha_{13,12,11}$	123.7	123.7
$C_{14,15}$	133.5	133.5	$\alpha_{8,13,12}$	118.2	118.3
$C_{15,16}$	139.6	139.6	$\alpha_{9,8,13}$	122.2	122.1
$C_{16,17}$	139.5	139.5	$\alpha_{19,6,5}$	120.4	120.6
$C_{17,18}$	139.2	139.2	$\alpha_{20,19,6}$	124.3	120.1
$C_{18,7}$	140.3	140.3	$\alpha_{21,20,19}$	110:6	110.5
$C_{6,19}$	146.9	147.7	$\alpha_{22,21,20}$	108.5	108.9
$C_{19,20}$	141.5	141.6	$\alpha_{23,22,21}$	106.8	106.4
$C_{20,21}$	139.6	138.6	$\alpha_{19,23,22}$	106.7	106.1
$C_{21,22}$	140.7	141.7	$\alpha_{24,22,21}$	125.3	124.1
$C_{22,23}$	149.7	150.7	$\alpha_{25,24,22}$	129.3	126.9
$C_{23,19}$	142.2	142.1	$\alpha_{26,25,24}$	128.3	130.7
$C_{22,24}$	139.3	140.1	$\alpha_{27,26,25}$	129.5	127.1
$C_{24,25}$	139.7	140.7	$\alpha_{28,27,26}$	129.1	132.3
$C_{25,26}$	139:8	139.2	$\alpha_{23,28,27}$	129.3	126.3
$C_{26,27}$	139.7	140.5	$\alpha_{19,23,28}$	126.8	127.2
$C_{27,28}$	139.9	139.8	$\tau_{9,8,4,3}$	1.3	0.2
$C_{28,23}$	139.3	141.7	$\tau_{13,8,4,5}$	1.8	0.6
$\alpha_{3,2,1}$	122.8	122.7	$\tau_{18,7,2,3}$	2.6	5.1
$\alpha_{4,3,2}$	118.2	118.2	$\tau_{14,7,2,1}$	3.2	5.8
$\alpha_{5,4,3}$	122.7	122.6	$\tau_{20,19,6,5}$	37.4	52.9
$\alpha_{6,5,4}$	119.8	119.7	$\tau_{23,19,6,1}$	39.6	52.0
$\alpha_{1,6,5}$	116.8	117.1	$\tau_{9,4,2,18}$	2.5	3.1

Table S2: Most dominant ($\psi_0 \rightarrow \psi_v$) contribution to TDDFT calculated electronic excitations for **4a**, **4b**.
ψ_0: occupied Kohn-Sham orbital, ψ_v: virtual Kohn-Sham orbital, A^2 = square of excitation amplitude

	**4a**						**4b**					
	B3LYP		CAMB3LYP		TPSSH		B3LYP		CAMB3LYP		TPSSH	
No.	$\psi_0 \rightarrow \psi_v$	A^2	$\psi_0 \rightarrow \psi_v$	A^2	$\psi_0 \rightarrow \psi_v$	A^2	$\psi_0 \rightarrow \psi_v$	A^2	$\psi_0 \rightarrow \psi_v$	A^2	$\psi_0 \rightarrow \psi_v$	A^2
I	94→95	0.98	94→95	0.96	94→95	0.98	106→107	0.95	106→107	0.91	106→107	0.96
II	94→96	0.86	94→96	0.66	94→97	0.97	106→108	0.40	106→108	0.63	106→108	0.01
	94→97	0.16	92→95	0.25			106→109	0.54	105→107	0.22	106→109	0.98
	92→95	0.09	94→98	0.08					106→110	0.11		
III	94→96	0.14	93→96	0.16	94→96	0.87	106→108	0.52	104→108	0.10	106→108	0.89
	94→97	0.82	94→97	0.69	92→95	0.05	106→109	0.43	106→109	0.75	106→110	0.06
IV	93→96	0.92	94→96	0.28	93→96	0.91	105→108	0.43	106→108	0.27	105→108	0.04
			92→95	0.37			104→108	0.20	105→107	0.42	104→108	0.61
			94→98	0.21			105→109	0.12	106→110	0.13	105→109	0.18
V	93→97	0.31	93→96	0.30	93→97	0.34	102→109	0.45	105→110	0.16	105→107	0.06
	90→97	0.41	92→96	0.22	92→96	0.25	104→109	0.25	106→110	0.10	104→109	0.77
	92→97	0.07	94→97	0.13	90→96	0.26	102→107	0.12	105→108	0.54		
	90→96	0.06	93→98	0.08								
VI	93→97	0.30	93→96	0.20	93→97	0.42	102→109	0.23	104→108	0.58	104→110	0.56
	90→97	0.27	92→96	0.43	92→96	0.20	104→109	0.50	106→109	0.16	105→110	0.12
	92→97	0.08	94→97	0.08	90→96	0.16	102→107	0.03	104→110	0.13		
	90→96	0.07	93→98	0.08								
VII	92→95	0.21			92→95	0.20	105→107	0.26			105→107	0.22
	90→95	0.09			94→98	0.13	106→110	0.15			106→110	0.12
	89→96	0.08					102→107	0.14			101→107	0.14

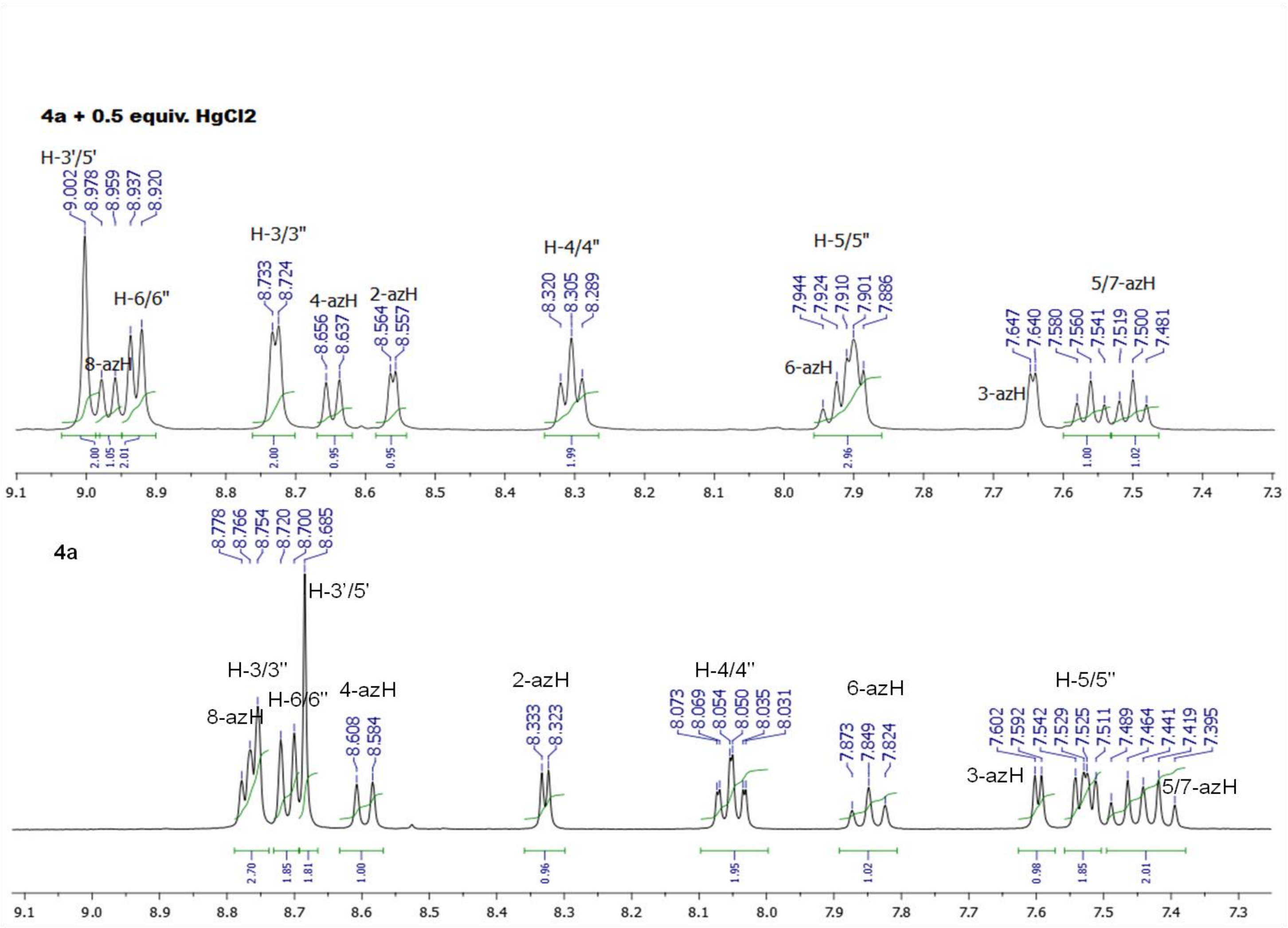

Figure S2: Overlay of the ^{1}H NMR spectra of 4′-azulenyl-2,2′:6′,2″-terpyridine (**4a**) and the corresponding Hg(II) complex in DMSO-d_6.
